# The impact of ethnicity on longitudinal humoral and cellular immune responses to SARS-CoV-2 booster vaccination

**DOI:** 10.1038/s41598-026-54217-5

**Published:** 2026-06-27

**Authors:** Christopher A. Martin, Joshua Nazareth, Lucy Teece, Daniel Pan, Sam Scott, Amar Jarkhi, Mrinal Das, Luke Bryant, Nisha George, Marjan Gohar, Valerie Renals, Victoria Wenn, Denny Vail, Aleesha Karia, Paul Moss, Brian J. Willett, Andrea Tattersall, Bassam Hallis, Ashley D. Otter, Cathy Rowe, Pranab Haldar, Andrea Cooper, Manish Pareek

**Affiliations:** 1 Division of Public Health and Epidemiology, School of Medical Sciences, University of Leicester, University Road, Leicester, LE1 7RH UK; 2 Department of Infection and HIV Medicine, University Hospitals of Leicester NHS Trust, Leicester, UK; 3 Development Centre for Population Health, University of Leicester, Leicester, UK; 4 Leicester NIHR Biomedical Research Centre, Leicester, UK; 5 Department of Population Health Sciences, College of Life Sciences, University of Leicester, Leicester, UK; 6 Li Ka Shing Centre for Health Information and Discovery, Oxford Big Data Institute, University of Oxford, Oxford, UK; 7 University of Glasgow Centre for Virus Research, University of Glasgow, Bearsden Road, Glasgow, UK; 8 Division of Microbiology and Infection, School of Biological and Biomedical Sciences, University of Leicester, Leicester, UK; 9 Research Space, University Hospitals of Leicester NHS Trust, Leicester, UK; 10 Institute of Immunology and Immunotherapy, University of Birmingham, Birmingham, UK; 11 Revvity (Certimmune), Abingdon, Oxon UK; 12 UK Health Security Agency, Porton Down, Salisbury, UK; 13 Division of Respiratory Sciences, School of Medical Sciences, University of Leicester, Leicester, UK; 14 Department of Respiratory Medicine, University Hospitals of Leicester NHS Trust, Glenfield Hospital, Leicester, UK; 15 NIHR Applied Research Collaboration East Midlands, Leicester, UK

**Keywords:** Diseases, Health care, Immunology, Medical research

## Abstract

Ethnic differences in SARS-CoV‑2 vaccine immunogenicity have been reported after primary vaccination, but it remains unclear whether these disparities persist following booster doses or whether patterns of immune boosting and waning differ across ethnic groups. In this longitudinal study of healthcare workers enrolled in the BE‑DIRECT cohort, we evaluated humoral and cellular immune responses before booster vaccination, 3–4 weeks afterwards, and at approximately six months. Anti‑spike and neutralising antibodies, as well as T‑cell ELISpot responses to spike peptides, were quantified, and changes across boosting and waning phases were examined using multivariable regression adjusting for demographic factors, vaccine regimen, prior infection and baseline immune measures. South Asian participants demonstrated substantially higher post‑booster anti‑spike titres than White participants, although titres converged by six months. After adjustment, South Asian individuals exhibited greater increases in total anti-spike and neutralising antibody titres, while decline in antibody titres between post-booster and six-month post-booster sampling points were similar across ethnic groups. T‑cell responses during the boosting phase did not differ, but decline of S2‑specific and overall spike‑specific T‑cell responses between post-booster and six-month post-booster sampling points occurred more slowly in South Asians. These findings indicate that South Asian healthcare workers mount stronger early booster‑induced antibody responses and exhibit more sustained T‑cell immunity, highlighting the need to understand underlying mechanisms and implications for vaccine effectiveness.

**Supplementary Information:**

The online version contains supplementary material available at https://doi.org/10.1038/s41598-026-54217-5.

## Introduction

COVID-19 has caused considerable morbidity and mortality and has disproportionately affected individuals from ethnic minority groups^1^ and healthcare workers (HCWs)^2,3^. Mass vaccination against SARS-CoV-2 has provided an effective means of protecting the population from severe COVID-19^4,5^. In recognition of their increased personal risk of COVID-19 and the risk of transmitting infection to patients under their care, HCWs have been prioritised in the UK vaccine programme^6^. However, vaccine induced immunity has been shown to be short-lived and may be evaded by new variants of SARS-CoV-2^7^ necessitating ‘booster’ vaccination which until recently has been offered annually to UK HCWs since 2021^8^.

It is now clear that those from ethnic minority groups are at higher risk of SARS-CoV-2 infection than majority groups which is likely to be mediated by greater exposure to the virus as a result of sociodemographic and economic inequities^1^. Individuals from ethnic minority groups may also be at higher risk of severe COVID-19, although this relationship is less clear and may be changing over time^9^.

Despite the differences in COVID-19 outcomes by ethnicity and the importance of vaccination in protection against severe COVID-19, there are relatively few studies that examine whether vaccine immunogenicity differs according to the ethnicity of the vaccinee. Previous work suggests that the initial doses of SARS-CoV-2 vaccine induced higher SARS-CoV-2 specific antibody responses, serum neutralising activity and T cell responses in South Asian HCWs as compared to White HCWs, particularly in the early phase after vaccination^10^. These findings, at least as they relate to antibody titres, are in-line with a different UK HCW cohort^11^ and a large observational cohort study in the UK general population^12^.

Evidence on whether these differences persist with repeated doses of vaccine and, critically, whether waning of the immune response to vaccination differs according to ethnicity, is limited. Two studies from the US (a country with a very different ethnic make-up and approach to classifying ethnic group to the UK^13^ have considered the impact of ethnicity on vaccine responses longitudinally, though not as their primary exposure of interest. Both studies concluded that there were no differences in antibody titres between the ethnic groups studied and neither examined neutralising activity or T cell responses to vaccine^14,15^. We therefore sought to add to the limited evidence base on ethnic differences in humoral and cellular responses to SARS-CoV-2 booster vaccination using data from BE-DIRECT, a longitudinal cohort study in the UK.

## Results

### Formation of the analysed sample

**Fig. 1:**
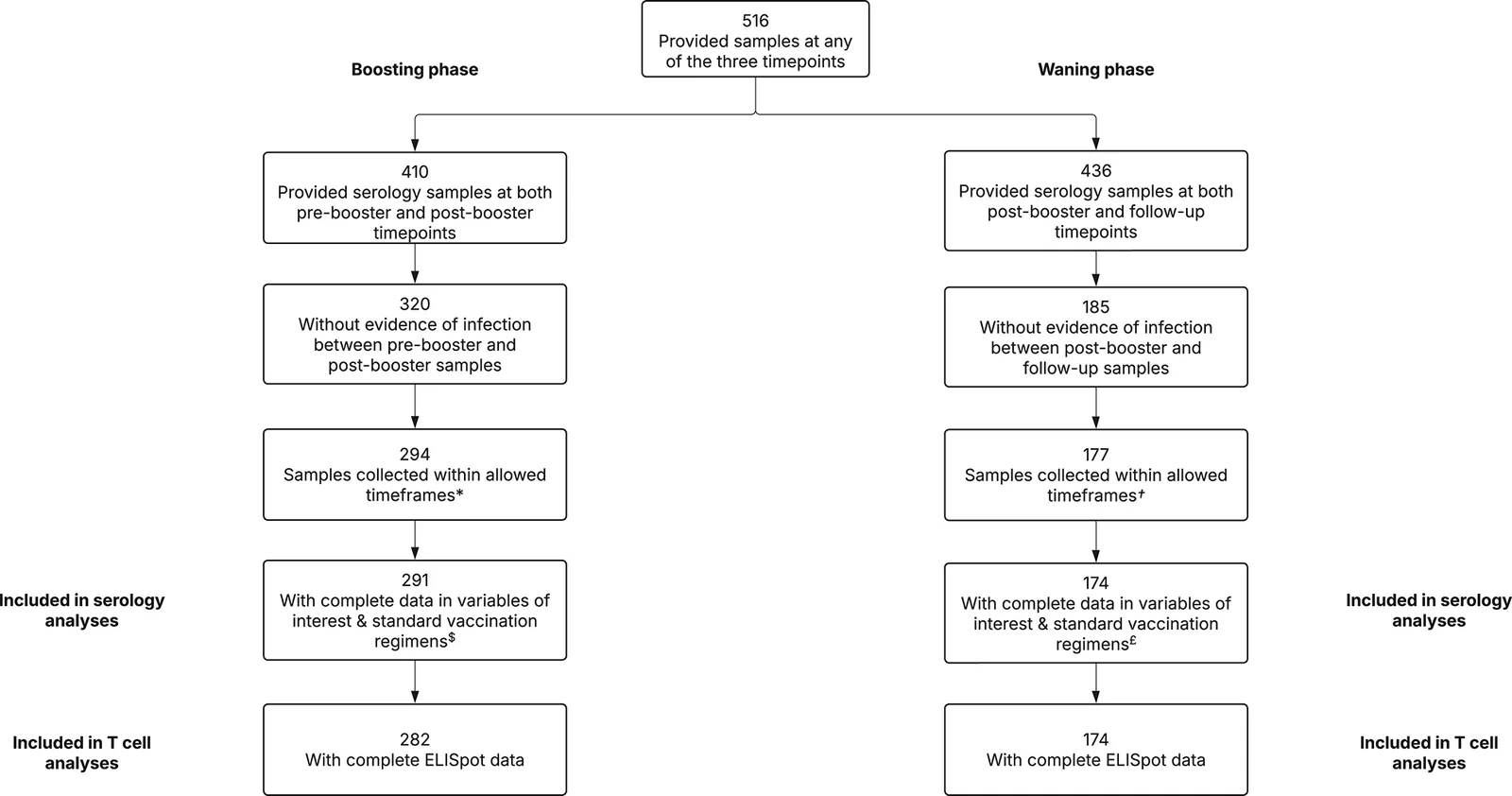
Formation of the analysed sample. *In the boosting phase those who did not receive vaccine within 21 days of pre-booster sample collection were excluded, as were those whose post-booster sample was collected earlier than 14 days or later than 50 days after vaccine. In the waning phase, those whose post-booster sample was collected earlier than 14 days or later than 50 days after vaccine were excluded. $ 3 additional exclusions were made in the neutralising activity analysis due to missing data for this outcome. £ 2 additional exclusions were made in the neutralising activity analysis due to missing data for this outcome.

Figure 1 shows the formation of the analysed cohort and the numbers of individuals included in each analysis. In total, 291 HCWs (241 [80.9%] White, 40 [13.4%] South Asian and 27 [5.7%] Black/Mixed/Other) were included in analysis of the boosting phase of the immune response and 174 (137 [78.7%] White, 28[16.1%] South Asian and 9 [5.2%] Black/Mixed/Other) were included in the analysis of the waning phase.

### Description of the cohort

Table 1 shows a description of the HCW cohorts included in the analysis of each phase of the immune response by ethnic group. Median age of those included in the analysis of the boosting phase was 45 (IQR 33.5–53.5) and the majority (245, 81.7%) were female. Overall, 232 (78.1%) received BNT vaccine for their initial two doses and 65 (21.9%) received ChAd. There were significant differences in the number of days between booster vaccination and sample collection by ethnicity (median [IQR] White: 23^21–27^; South Asian 26^23–29^; Black/Mixed/Other 26^21–31^. There were no significant differences by ethnicity in time interval between completing the primary course of vaccination and receiving booster vaccination. Those from South Asian groups were significantly more likely than White HCWs to report having physical contact with COVID-19 patients in the baseline survey.

**Table 1:** Description of the cohort included in the analysis of each phase of the immune response to SARS-CoV-2 vaccination by ethnicity.

Boosting phase	Overall (*n*=291)	White (*n*=235)	South Asian (*n*=40)	Black/mixed/other (*n*=16)	*P* value*
Age in years, med (IQR)	45 (34 – 54)	47 (34 – 54)	38 (32.5 – 49)	42 (30 – 46)	0.04
Sex					
Male	53 (18.3)	40 (17.4)	9 (22.5)	4 (23.5)	
Female	238 (81.7)	195 (82.6)	31 (77.5)	12 (76.5)	0.55
Vaccine regimen					
BNT/BNT/BNT	226 (77.9)	178 (75.7)	35 (87.5)	13 (86.7)	
ChAd/ChAd/BNT	64 (22.1)	57 (24.3)	5 (12.5)	2 (13.3)	0.18
Days between booster vaccine and post-booster sample collection, med (IQR)	23 (21 – 27)	22 (21 – 27)	26 (23 – 29)	26.5 (21.5 – 31)	0.002
Pre-booster anti-nucleocapsid antibody status					
Negative	217 (74.6)	179 (76.2)	28 (70.0)	10 (62.5)	0.37
Positive	74 (25.4)	56 (23.8)	12 (30.0)	6 (37.5)	
Time in weeks between completion of primary vaccination course and booster vaccine, med(IQR)	28.1 (27 – 30.7)	28 (27 – 30.4)	28.4 (27.1 – 32.4)	28.9 (26.7 – 32)	0.37

Waning phase	Overall (*n*=174)	White (*n*=137)	South Asian (*n*=28)	Black/mixed/other (*n*=9)	*P* value*
Age in years, med (IQR)	49 (38 – 56)	51 (40 – 56)	45.5 (36.5 – 52.5)	48 (29 – 56)	0.20
Sex					
Male	30 (17.2)	24 (17.5)	6 (21.4)	0 (0.0)	0.33
Female	144 (82.8)	113 (82.5)	22 (78.6)	9 (100.0)	
Vaccine regimen					
BNT/BNT/BNT	133 (77.3)	105 (77.2)	20 (71.4)	8 (100.0)	0.23
ChAd/ChAd/BNT	39 (22.7)	31 (22.8)	8 (28.6)	0 (0.0)	
Days between post-booster and follow-up sample collection, med (IQR)	189 (178 – 218)	188 (177 – 213)	211.5 (186 – 238.5)	204 (183 – 218)	0.01
Post-booster anti-nucleocapsid antibody status					
Negative	100 (57.5)	83 (60.6)	12 (42.9)	5 (55.6)	0.22
Positive	74 (42.5)	54 (39.4)	16 (57.1)	4 (44.4)	

*By chi-squared for categorical variables and Kruskal-Wallis for continuous variables.

For those included in the analyses of the waning phase of the immune response, median age was 49 (38–56) and 144 (82.8% were female). Significant differences in time between the post-booster and follow-up sample collection were found between ethnic groups (median [IQR] White: 188 [177–213]; South Asian 211.5 [186–238.5]; Black/Mixed/Other 204 [183–218]).

### Humoral responses to SARS-CoV-2 vaccination

**Fig. 2:**
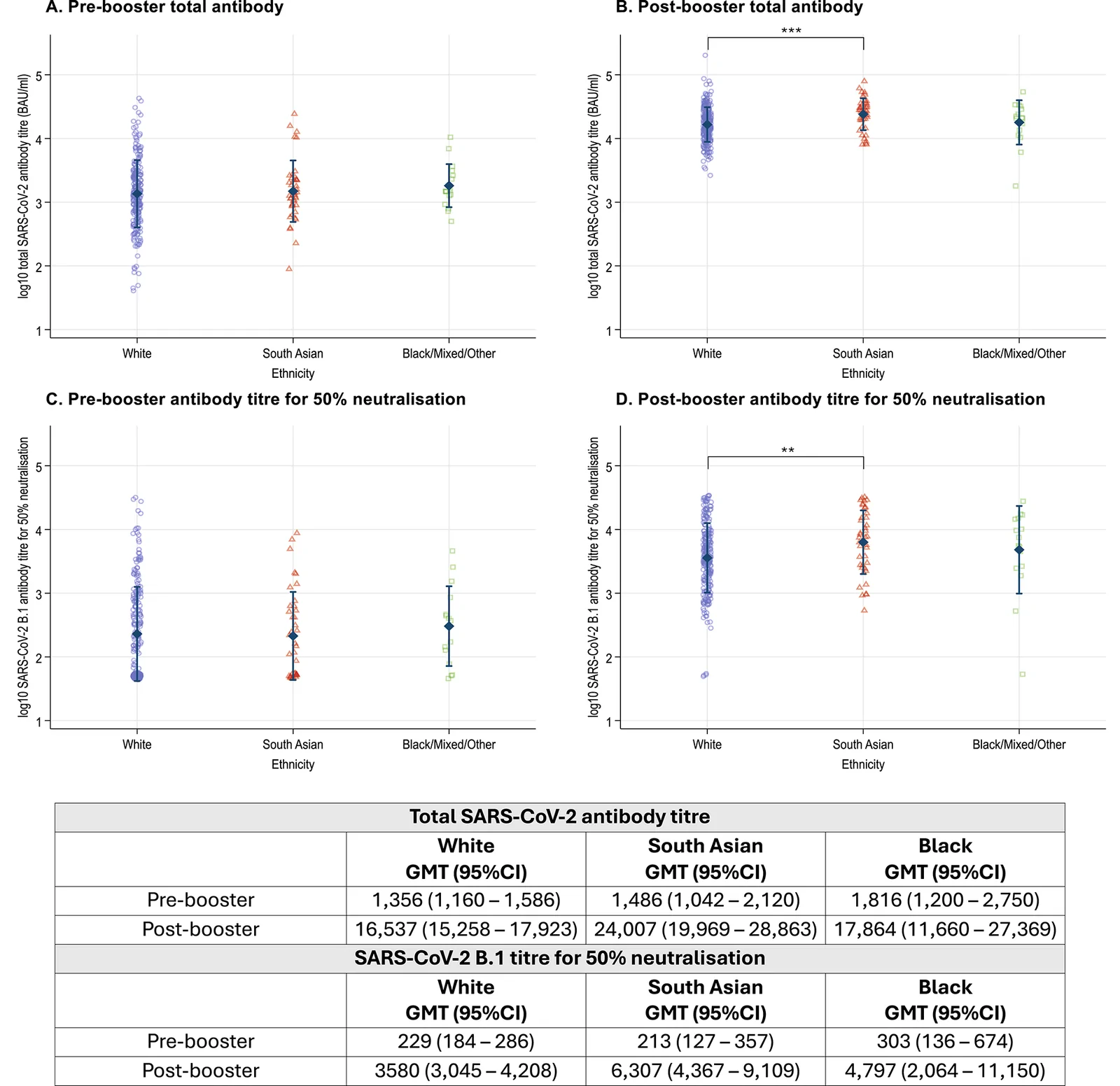
Comparison of unadjusted total SARS-CoV-2 anti-spike titre and the titre for 50% neutralisation of SARS-CoV-2 B.1 by ethnicity during the bosting phase of the immune response. Figure shows total SARS-CoV-2 anti-spike antibody titres and titres for 50% neutralisation of SARS-CoV-2 B.1 in the pseudotype neutralisation assay stratified by ethnic group (White – blue circles, South Asian – red triangles and Black/Mixed/Other – green squares) for those included in analyses of the boosting phase. The table shows geometric mean titre and 95% confidence interval for each timepoint according to phase of the immune response.

**Fig. 3:**
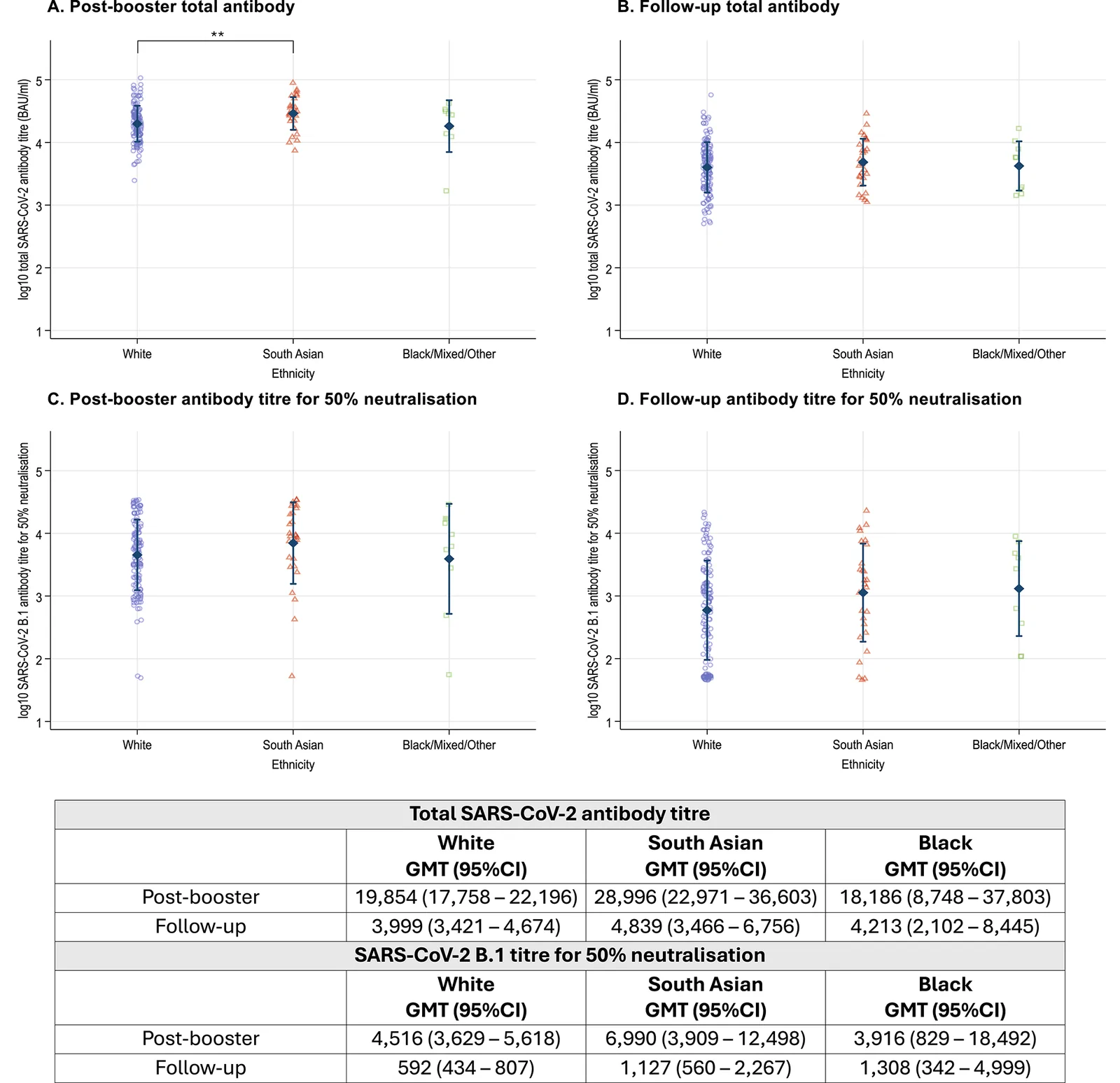
Comparison of unadjusted total SARS-CoV-2 anti-spike titre and the titre for 50% neutralisation of SARS-CoV-2 B.1 by ethnicity during the waning phase of the immune response. Figure shows total anti-spike antibody titres and titres for 50% neutralisation of SARS-CoV-2 B.1 in the pseudotype neutralisation assay stratified by ethnic group (White – blue circles, South Asian – red triangles and Black/Mixed/Other – green squares) for those included in analyses of the waning phase. The table shows geometric mean titre and 95% confidence interval for each timepoint according to phase of the immune response.

Unadjusted antibody titres for each phase of the immune response by ethnic group are shown in Figs. 2 and 3. Post-booster anti-spike antibody titres were higher in South Asian HCWs than in White HCWs 24,007 BAU/ml (95%CI 19,968–28,863 BAU/ml) vs. 16,574 BAU/ml (95%CI 15,258–17,923 BAU/ml) *p* < 0.001. There were no significant differences in pre-booster or follow-up titres by ethnicity. Unadjusted GMRs for the relationship of covariates with post-booster and follow-up total anti-spike antibody titres are shown in Supplementary Table 2.

### Boosting phase of humoral response

**Fig. 4:**
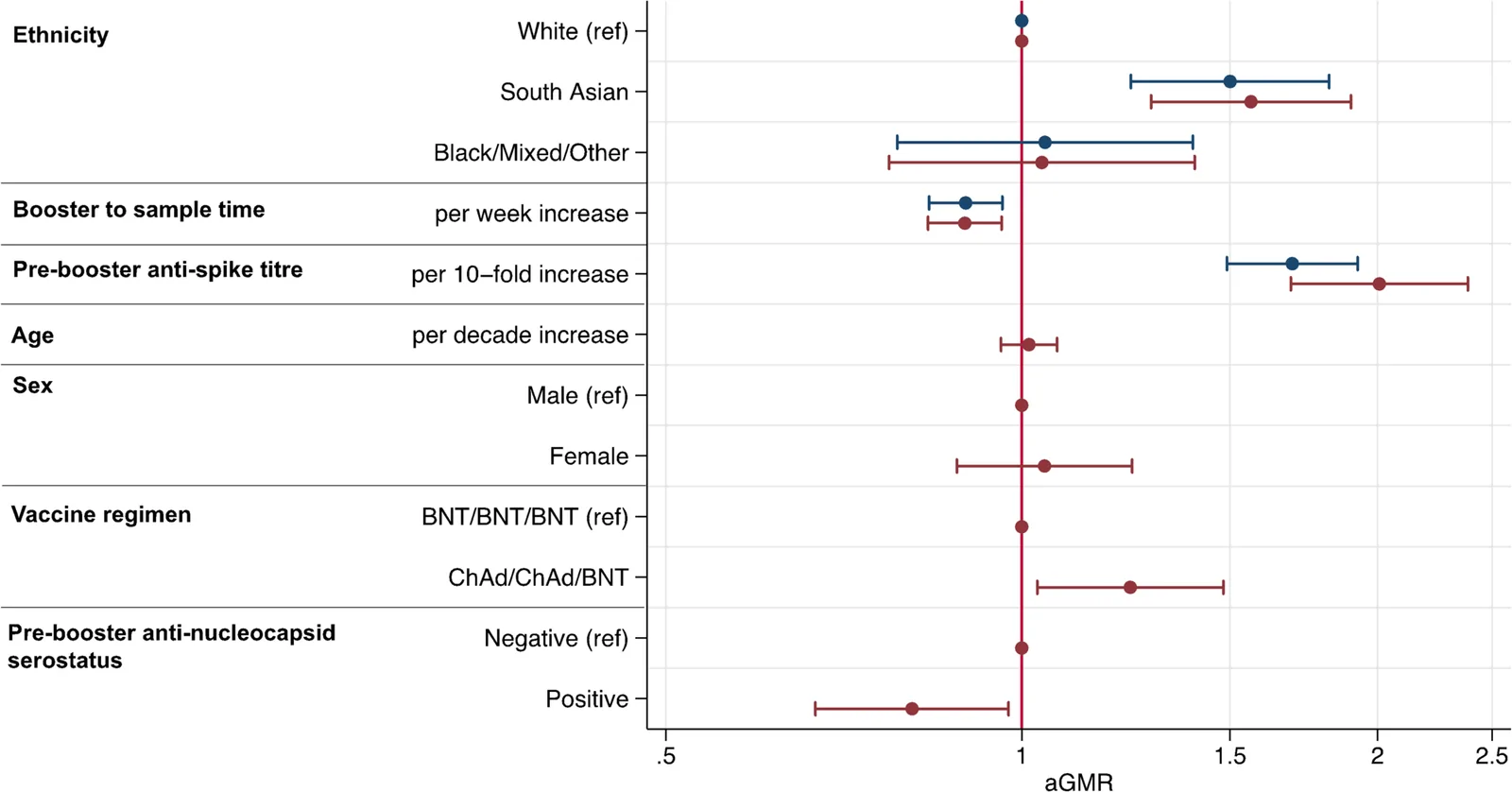
Linear regression models to show the relationship between ethnicity and other sociodemographic and vaccine related parameters with post-booster SARS-CoV-2 total anti-spike antibody titre. Figure shows the results of two linear regression models. The outcome is log10 transformed antibody titres at the post-booster visit – around 3–4 weeks after vaccination. Coefficients were exponentiated and presented as adjusted geometric mean ratio (aGMR) and 95% confidence interval (95%CI). Blue dots and spikes show results from the ‘minimally adjusted’ model and red dots and spikes show results from the ‘fully adjusted’ model which is additionally adjusted for the listed pre-specified confounders. One participant had missing data for vaccine schedule and is therefore missing from the fully adjusted model.

In a multivariable linear regression model adjusted for time between booster and sample collection and pre-booster total SARS-CoV-2 antibody titre, log_10_-post-booster aGMR was 1.50 (95% CI 1.24–1.81, *p* < 0.001) for South Asian HCWs compared to White. No differences were found between the White and the Black/Mixed/Other group (*p* = 0.47). Estimates did not materially change after adjustment for confounders (see Fig. 4 and Supplementary Table 3). Similar results were obtained when analysis was restricted to those without serological evidence of previous infection (Supplementary Table 4). A vaccination regimen of ChAd/ChAd/BNT was found to elicit a greater change in antibody titres than a BNT/BNT/BNT regimen (aGMR 1.24, 95%CI 1.04–1.49, *p* = 0.02).

**Fig. 5:**
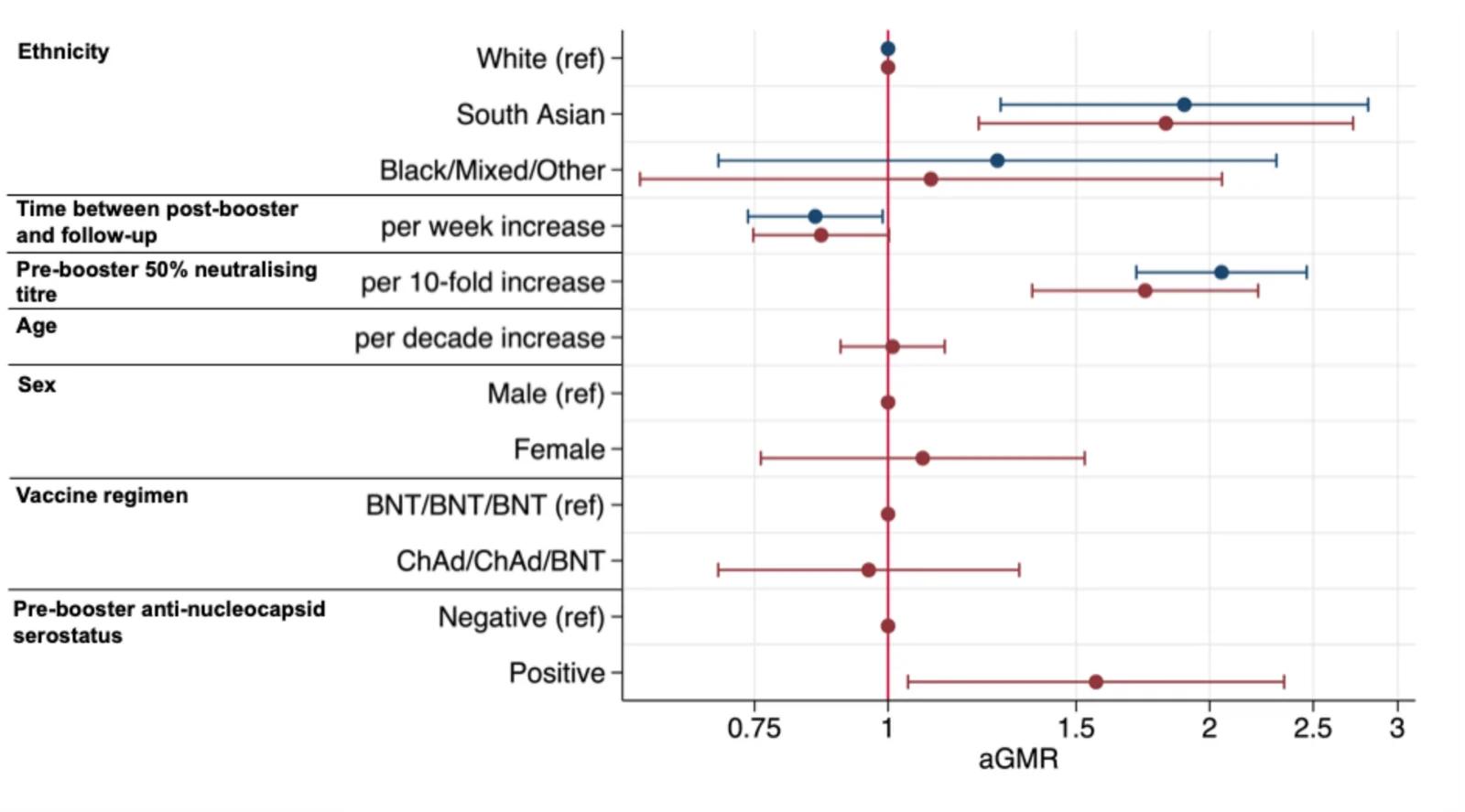
Linear regression models to show the relationship between ethnicity and other sociodemographic and vaccine related parameters with post-booster titre for 50% neutralisation of SARS-CoV-2 B.1. Figure shows the results of two linear regression models. The outcome is log10 transformed titres for 50% neutralisation of SARS-CoV-2 B.1 in the pseudotype neutralisation assay at the post-booster visit – around 3–4 weeks after vaccination. Coefficients were exponentiated and presented as adjusted geometric mean ratio (aGMR) and 95% confidence interval (95%CI). Blue dots and spikes show results from the ‘minimally adjusted’ model and red dots and spikes show results from the ‘fully adjusted’ model which is additionally adjusted for the listed pre-specified confounders. One participant had missing data for vaccine schedule and is therefore missing from the fully adjusted model.

In the analysis of neutralising antibody titres, South Asian HCWs were also demonstrated to have a more robust response to vaccination than White HCWs (aGMR 1.82, 95%CI 1.22–2.72, *p* = 0.002) with the estimates only negligibly affected by additional adjustments for confounders (Fig. 5 and Supplementary Table 5. Similar results were obtained when analysis was restricted to those without serological evidence of previous infection (Supplementary Table 4).

Supplementary Table 6. shows the analysis of absolute and fold change in total antibody titres. Fold change in titre was over 1.5 times higher in South Asian HCWs compared to White HCWs (1.62, 95%CI 1.28–2.05 for absolute change; 1.80, 95% CI 1.35–2.40 for fold change in the fully adjusted models).

### Waning phase of humoral response

**Fig. 6:**
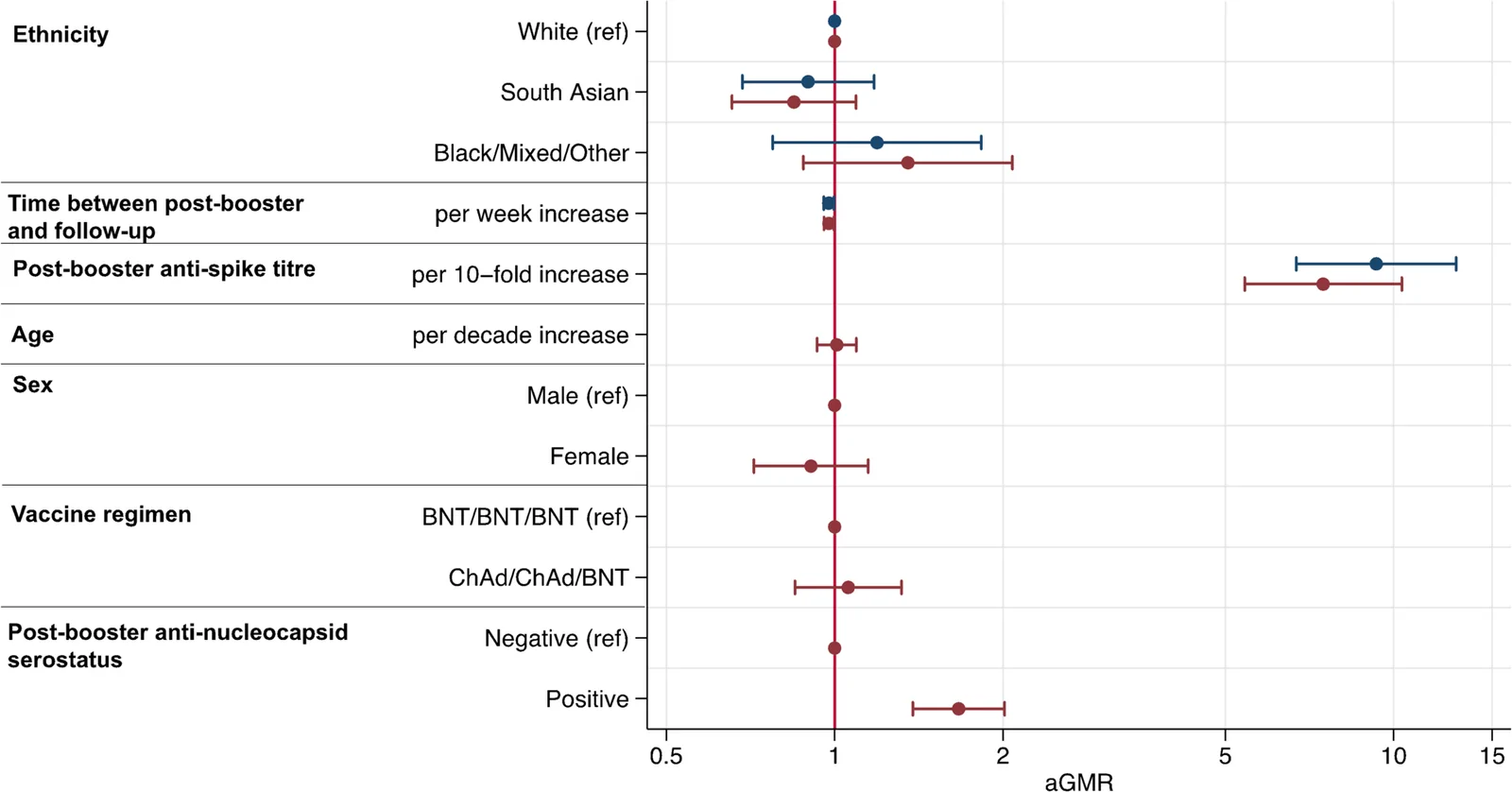
Linear regression models to show the relationship between ethnicity and other sociodemographic and vaccine related parameters with follow-up SARS-CoV-2 total anti-spike antibody titre. Figure shows the results of two linear regression models. The outcome is log10 transformed follow-up antibody titres – around 6 months after booster vaccination. Coefficients were exponentiated and presented as adjusted geometric mean ratio (aGMR) and 95% confidence interval (95%CI). Blue dots and spikes show results from the ‘minimally adjusted’ model and red dots and spikes show results from the ‘fully adjusted’ model which is additionally adjusted for the listed pre-specified confounders. One participant had missing data for vaccine schedule and is therefore missing from the fully adjusted model.

**Fig. 7:**
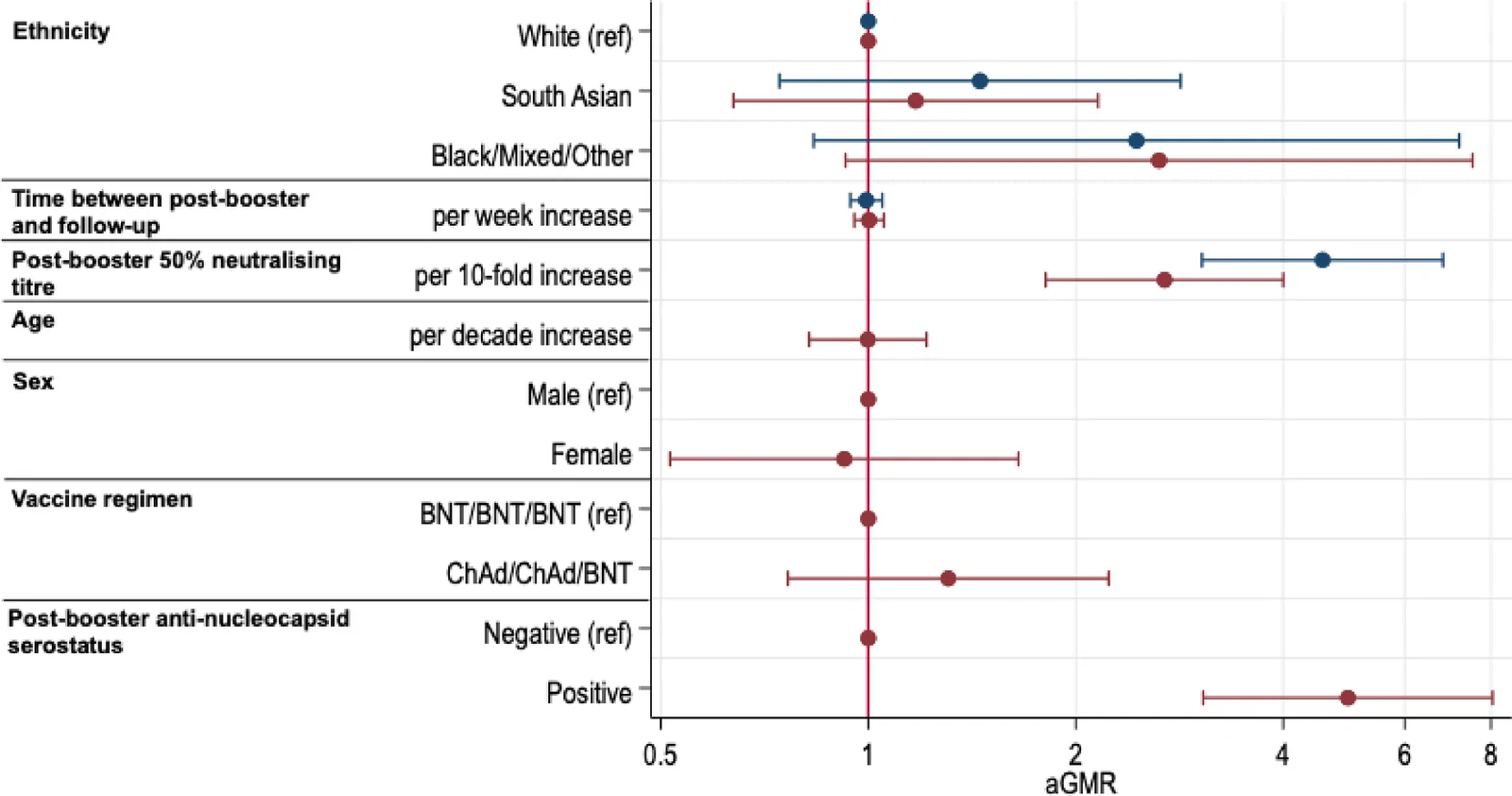
Linear regression models to show the relationship between ethnicity and other sociodemographic and vaccine related parameters with follow-up titre for 50% neutralisation of SARS-CoV-2 B.1. Figure shows the results of two linear regression models. The outcome is log10 transformed titres for 50% neutralisation of SARS-CoV-2 B.1 in the pseudotype neutralisation assay at the follow-up timepoint – around 6 months after booster vaccination. Coefficients were exponentiated and presented as adjusted geometric mean ratio (aGMR) and 95% confidence interval (95%CI). Blue dots and spikes show results from the ‘minimally adjusted’ model and red dots and spikes show results from the ‘fully adjusted’ model which is additionally adjusted for the listed pre-specified confounders. One participant had missing data for vaccine schedule and is therefore missing from the fully adjusted model.

There were no significant ethnic differences in the change in total SARS-CoV-2 anti-spike antibody titres between the post-booster and follow-up timepoints in the minimally or fully adjusted models (Fig. 6 and supplementary Table 7). Post-booster antibody titre had a strong positive association with follow-up titre (aGMR 7.48, 95%CI 5.41–10.34, *p* < 0.001, per 10-fold change in post-booster titre). Those who were anti-nucleocapsid seropositive at their post-booster visit had a more sustained antibody response to vaccination (aGMR 1.67, 1.38–2.01, *p* < 0.001). There were also no differences in the change in neutralising antibody titres between timepoints (Fig. 7 and Supplementary Table 8).

Results from the analysis of absolute and fold change in titre between the post-booster and follow-up visits are shown in Supplementary Table 9. South Asian HCWs have a higher absolute change in titre during the waning phase of the immune response (adjusted ratio 1.60, 95%CI 1.20–2.13, *p* = 0.001), however, there were no ethnic differences in fold change in antibody titre.

### Sensitivity analysis

Additional adjustment of the multivariable models of the humoral vaccine response in the boosting and waning phase for occupational exposure (whether a HCW reported physical contact with COVID-19 patients or not) had a negligible effect on the relationship between ethnicity and antibody titre (Supplementary Table 10).

### Cellular responses to SARS-CoV-2 vaccination

Unadjusted ELISpot counts in response to SARS-CoV-2 spike peptides are shown in Supplementary Fig. 1 and Supplementary Table 11. Median ELISpot counts at the post-booster timepoint in response to each of the three peptide panels (S1, S2 and spike) was higher in White HCW than in South Asian HCW though this difference was not statistically significant. Median ELISpot counts in response to S2 and spike peptides at the follow up timepoint was marginally higher in South Asian than White HCW though this was significant only for S2 (South Asian: 5, IQR 2.5–9 vs. White: 3, IQR 1–7, *p* = 0.04).

### Boosting phase of T cell response

**Fig. 8:**
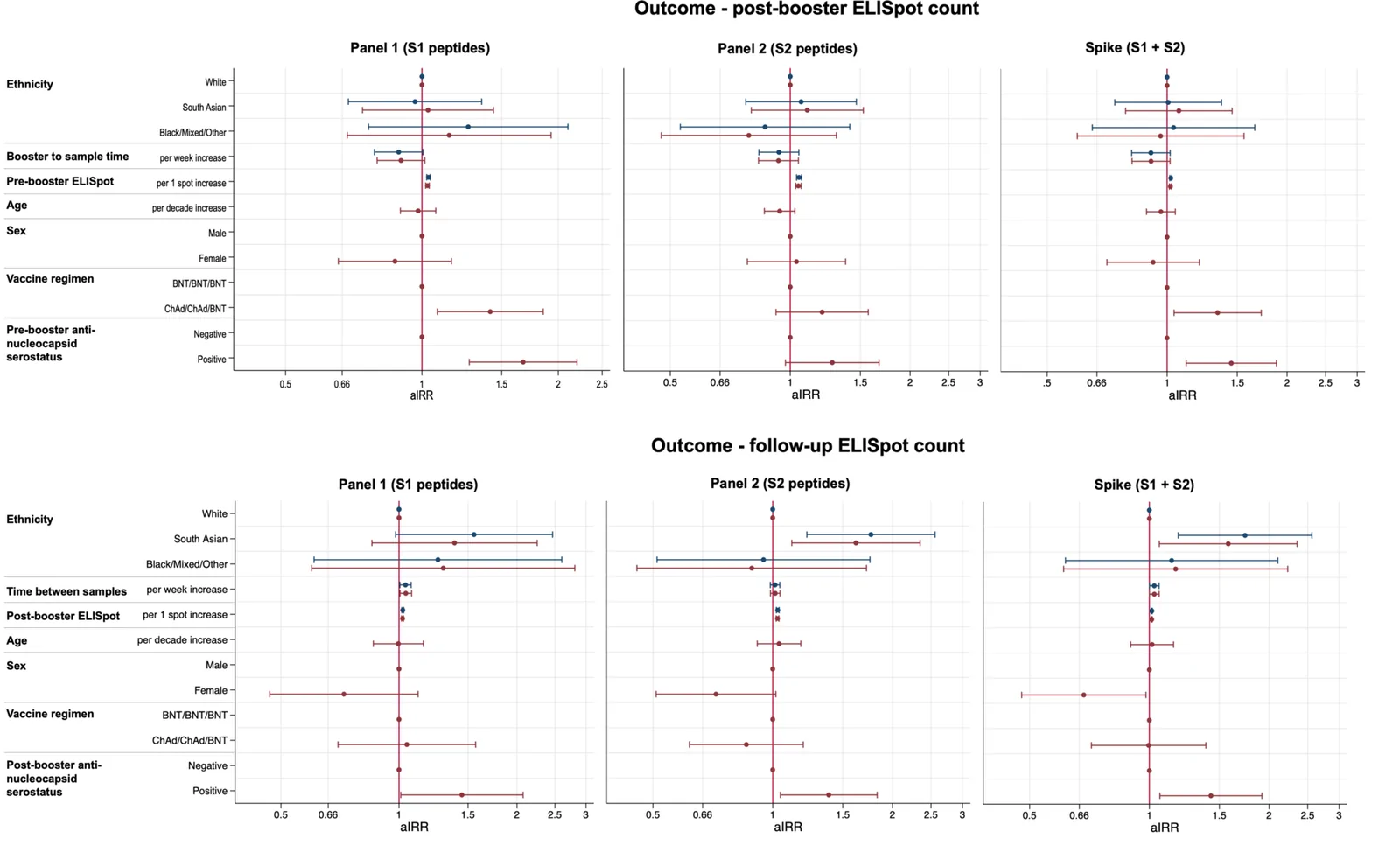
Negative binomial regression models to show the relationship between ethnicity and other sociodemographic and vaccine related parameters with post-booster and follow-up T cell ELISpot counts. Figure shows the results of multiple negative binomial regression models. The outcome is T cell ELISpot count at the post-booster appointment (top row) and the follow-up appointment (bottom row). Coefficients were exponentiated and presented as adjusted incidence rate ratios (aIRR) and 95% confidence interval (95%CI). Blue dots and spikes show results from the ‘minimally adjusted’ model and red dots and spikes show results from the ‘fully adjusted’ model which is additionally adjusted for the listed pre-specified confounders. One participant had missing data for vaccine schedule and is therefore missing from the fully adjusted model.

The results of the multivariable negative binomial regression examining changes in post-booster ELISpot count are shown in Fig. 8. Ethnicity was not found to be associated with changes in ELISpot count in the boosting phase. In the fully adjusted model, responses to S1 and spike (S1 + S2) peptides were higher in those who received ChAdOx1-S vaccine in their initial doses compared to those who received BNT162b2 (S1: aIRR 1.42, 95%CI 1.09–1.85, *p* = 0.01; spike: aIRR 1.34, 95%CI 1.04–1.72, *p* = 0.02). Those seropositive for anti-nucleocapsid antibody at their pre-booster appointment also had higher ELISpot counts in response to S1 and spike peptides (S1: aIRR 1.67, 95%CI 1.27–2.20, *p* < 0.001; spike: 1.45, 95%CI 1.12–1.88, *p* = 0.004).

### Waning phase of T cell response

ELISpot responses to S2 and spike peptides waned more slowly in South Asian ethnic groups compared to those from White ethnic groups in the minimally and fully adjusted model (fully adjusted S2 : aIRR 1.62, 95%CI 1.12–2.35, *p* = 0.01, fully adjusted spike: 1.58, 95% CI 1.06–2.35, *p* = 0.02). Those seropositive for anti-nucleocapsid antibody at their post-booster appointment had higher ELISpot responses to all peptide panels at follow-up compared to those who were seronegative (S1: aIRR 1.45, 95%CI 1.01–2.07, *p* = 0.04; S2: aIRR 1.38, 95%CI 1.05–1.83, *p* = 0.01; spike: aIRR 1.43, 95%CI 1.06–1.92, *p* = 0.03) (Fig. 8).

## Discussion

In this large longitudinal immunology cohort study, we found clear evidence that the initial response to the first SARS-CoV-2 booster vaccination is stronger in South Asian HCWs as compared to White HCWs, with peak total antibody titres around 1.5 times higher in the former group. We also found a similar relationship between ethnicity and SARS-CoV-2 B.1 neutralising titres. Importantly, after adjustment for peak antibody titre, there were no ethnic differences in SARS-CoV-2 antibody titres at 6 months. Our results indicate that absolute change in antibody titre over the waning phase of the serologic response is higher in South Asian HCWs compared to White, and that this is likely to be driven by the higher peak antibody titres in the former group. T cell responses to SARS-CoV-2 S2 and spike peptides wane more slowly after booster vaccination in South Asian HCWs compared to White HCWs.

The higher post-booster total anti-SARS-CoV-2 antibody titres in South Asian HCWs as compared to White HCWs demonstrated in this study are consistent with studies conducted during the initial vaccine roll-out, including work from this cohort which showed that early (between 14 and 50 days after second vaccine dose) humoral responses to the second dose of SARS-CoV-2 vaccination were higher amongst South Asian HCWs compared to White HCWs^10^. Higher serologic responses to two doses of SARS-CoV-2 vaccine in those from Asian ethnic groups have also been demonstrated in a cohort of patients with immunosuppressive disease^16^. This longitudinal analysis builds upon this previous work by establishing firstly, that these differences persist with repeated vaccination, secondly, assuming that we captured the peak antibody titre after vaccination, that there are differences in the change in antibody titre in the early phase after vaccination and thirdly, that a similar relationship to that observed with total SARS-CoV-2 antibody titres exists between ethnicity and functional SARS-CoV-2 B.1 antibody titres.

To our knowledge, this is the first study to investigate the waning phase of the humoral response to SARS-CoV-2 booster vaccination by ethnicity. Whilst we determined that the absolute decline in antibody titre between the post-booster and follow-up timepoint was greater in South Asian ethnic groups than White groups, we demonstrated no differences between these groups when analysing fold change in titre or in analysis of follow-up titre after adjustment for post-booster titre. Differences in absolute change in titre over the 6 months between post-booster and follow-up sample collection are therefore likely to be mediated through the strong positive correlation between post-booster titre (demonstrated to be higher in South Asian HCW than White) and follow-up titre, explaining why no ethnic differences are seen once post-booster titre is adjusted for.

We found that T cell responses to S2 and spike peptides after booster vaccination wane more slowly in South Asian as compared to White HCWs. Interestingly, we did not find ethnic differences in the early period after vaccination as we previously demonstrated in the response to the initial doses of SARS-CoV-2 vaccine^10^. Our work represents the first longitudinal investigation of the impact of ethnicity on T cell responses to SARS-CoV-2 booster vaccination, although a previous cross sectional examination of HCWs with laboratory confirmed SARS-CoV-2 infection sampled 16–18 weeks after UK ‘lockdown’ found no ethnic differences in T cell responses to a variety of SARS-CoV-2 peptide panels^17^. There is evidence to suggest that cellular responses to SARS-CoV-2 vaccination make a substantial contribution to protection against severe disease and may have greater influence over protection against SARS-CoV-2 variants of concern than antibody responses^18,19^. An examination of vaccine effectiveness by ethnicity (perhaps by secondary analysis of existing trial data) is therefore warranted, to add to the limited information in the literature^20^. It is plausible that a more robust humoral response and a longer-lived T cell response in South Asian vaccinees could explain some of the reduction in the differences in risk of severe COVID-19 over time in this group compared to the White majority^1,9^.

The mechanisms underlying ethnic differences in SARS-CoV-2 vaccine immunogenicity have yet to be elucidated, but given the complexity of ethnicity as a construct which takes into account many facets of one’s life (e.g. language, diet, religion, ancestry^21^, are likely to be multifaceted. For example, recent work has highlighted the importance of human leukocyte antigen (HLA) alleles in serologic response to SARS-CoV-2 vaccine immunogenicity^22^, the frequency of particular HLA alleles is known to vary according to the ethnicity of the studied population and it is possible that such differences could mediate differences in immunogenicity of vaccines by ethnicity. Other genetic factors such as polymorphisms in genes related to antibody production have been shown to both influence vaccine induced anti-influenza A antibody repertoires and to vary by ethnicity^23^. Gut microbiota composition has been shown to relate to SARS-CoV-2 vaccine immunogenicity^24^ and also to differ according to ethnic group^25^(perhaps mediated through differences in diet, health and sociodemographic factors). Future research in this area should aim to understand the influence of these factors on humoral and cellular response to SARS-CoV-2 vaccination.

Our work has several limitations. A significant number of HCWs were infected between the post-booster and follow-up timepoints during the wave of infection driven by the emergence of the omicron variant of SARS-CoV-2. This reduced sample size and limited the power of statistical analysis of the waning phase. We do not have a large enough sample to test interaction terms in the multivariable models, such as the interaction of ethnicity and previous infection. We suggest this is a focus of larger studies in the future. By excluding those that became infected, and considering that higher post-booster antibody titres may protect against subsequent breakthrough infection, we may have selected for ‘high responders’ to vaccination in the analysis of the waning phase. The limited numbers of HCWs from ethnic groups other than White and South Asian necessitated the creation of a highly heterogenous third ethnicity category (Black/Mixed/Other) we did this in order to prevent exclusion of participants based on their ethnicity and, by presenting these data, to highlight that study of vaccine immunogenicity in cohorts with larger numbers of those from Black, Mixed and Other ethnic groups are urgently required. It is possible that the ethnic differences in post-booster vaccine responses observed in our study could be explained by differences in time to peak post-vaccine antibody titre by ethnicity. Studies in which participants from different ethnic groups are sampled more frequently in the early period after vaccination are needed to establish whether any such differences may have impacted our results.

In conclusion, we demonstrate that the higher anti-SARS-CoV-2 antibody titres achieved by South Asian vaccinees in comparison to White vaccinees after the initial doses of vaccine are also evident after administration of the first booster vaccine and that the degree of change in titre is also greater in the former group. Once these higher peak titres are accounted for, we do not find evidence to suggest that waning of the humoral response is different according to ethnicity. T cell responses to booster vaccination may be more durable in the South Asian cohort than the White cohort. Further work should be focussed on establishing the mechanisms that underlie these differences and whether they translate to ethnic differences in vaccine effectiveness.

## Methods

### Overview

The BE-DIRECT study is a longitudinal, immunology cohort study which recruited HCWs (including ancillary workers) aged 16 or over and working in Leicester, UK or the surrounding area. Participants were initially recruited into two separate cohort studies (the BELIEVE and DIRECT studies) which have been described in previous work^10,26^. We sought consent for further follow up from all participants of these studies and also supplemented recruitment by directly advertising the study to HCWs awaiting SARS-CoV-2 vaccine administration in the ‘vaccination hub’ at Leicester Royal Infirmary. All participants provided informed, written consent.

### Study visits

Participants who received a SARS-CoV-2 booster vaccination in the Autumn/Winter 2021–2022 season were invited to attend for the following study visits:

1. Pre-booster visit – blood sample and electronic questionnaire collected prior to vaccine administration (as close to vaccination date as practical for participant).
2. Post-booster visit – blood sample collected approximately 3–4 weeks after booster vaccine was administered.
3. Follow-up visit – blood sample and electronic questionnaire collected approximately 6 months after booster vaccination.

### Questionnaire data

We collected information on self-reported ethnicity (participants could select an ethnic group corresponding to the 18 Office for National Statistics [ONS] ethnic groups^27^, age, sex, type of SARS-CoV-2 vaccine received (BNT162b2 [Pfizer-BioNTech] or ChAdOx1-S [Oxford-AstraZeneca], hereafter referred to as BNT162b2 and ChAdOx1 respectively) in the initial vaccine roll out and the booster programme and the dates of receipt.

### Laboratory methods

In all laboratory assays described below, those performing the assays were blind to all the demographic and vaccine related parameters of the participants.

### SARS-CoV-2 serology assays

Anti-spike and anti-nucleocapsid SARS-CoV-2 serology were performed at UKHSA Porton Down on serum samples using the Roche Elecsys anti-SARS-CoV-2 S (Product code: 09203079190) and Roche Elecsys anti-SARS-CoV-2 (Product code: 09289275190) assays, respectively. Samples were considered positive for anti-spike antibodies if ≥ 0.8 BAU/ml, and positive for anti-nucleocapsid antibodies if ≥ 1 COI.

### SARS-CoV-2 pseudotype neutralisation assay

Plasmid constructs and 293-ACE2 cells were made at the Centre for Virus Research, Glasgow, UK as described previously^28–30^. Sera were screened for neutralising activity against HIV(SARS-CoV-2) pseudotypes bearing the spike glycoprotein of the ancestral lineage B.1 and bearing D614G^28,29^–chosen to reflect that the mRNA vaccines used in the 2021–2022 UK booster programme code for ancestral spike protein. Neutralising activity in each sample was measured by a serial dilution approach. Each sample was serially diluted in triplicate from 1:50 to 1:36450 in complete Dulbecco’s Modified Eagle Medium (DMEM) prior to incubation with approximately 1 × 10^6^ CPS per well of HIV (SARS-CoV-2) pseudotypes, incubated for 1 h, and plated onto 239-ACE2 target cells. After 48–72 h, luciferase activity was quantified by the addition of Steadylite Plus chemiluminescence substrate and analysis on a Perkin Elmer EnSight multimode plate reader (Perkin Elmer, Beaconsfield, UK). Antibody titre was then estimated by interpolating the point at which infectivity had been reduced to 50% of the value for the no serum control samples.

### SARS-CoV-2 ELISpot assay

To quantify T cell responses, we used T-SPOT^®^ *Discovery* SARS-CoV-2 platform (Oxford Immunotec), which use ELISpot technology to detect IFN-γ release from immune cells after exposure to SARS-CoV-2 peptides. This test is similar in methodology to the T-SPOT^®^.*TB* test which identifies patients infected with *M. tuberculosis*, and has been widely used clinically.

A peripheral venous blood sample of 6 mL was collected from participants and placed in a test tube of heparin anticoagulant. Peripheral blood mononuclear cells were isolated within 32 h of test performance. The T-SPOT *Discovery* SARS-CoV-2 test was performed according to the instructions of the kit. In brief, the peripheral blood mononuclear cells were counted, normalised and 250,000 PBMCs were plated into each well of a T-SPOT^®^ *Discovery* SARS-CoV-2 plate. Four different but overlapping peptides pools to cover protein sequences of SARS-CoV-2 - Spike 1 (S1), Spike 2 (S2), Nucleocapsid and membrane plus negative and positive controls were used (for further details see Supplementary Table 1). Cells were incubated overnight (16 to 20 h) at 37 °C with 5% CO_2_, washed with phosphate-buffered saline, and developed using an anti-IFN-γ antibody conjugate and substrate to detect the presence of secreted IFN-γ. Spot-forming cells (SFCs) were counted with an automated spot reader (Cellular Technology Ltd). As our analysis focussed on immune responses to vaccination, we present responses to the spike peptides S1, S2 and spike (S1 + S2).

### Statistical analysis

Our focus was on longitudinal responses to booster vaccination. We therefore excluded from the current analysis any participant who was unvaccinated or who did not elect to receive a booster vaccination.

We analysed immune response to booster vaccination into two phases:

1. A ‘boosting’ phase between pre-booster and post-booster timepoints where immune responses were assumed to be increasing to a peak at the time of the post-booster visit.
2. A ‘waning’ phase between the post-booster visit and the follow-up visit.

Participants with complete data for the two timepoints that make up one of these phases were included in the relevant analyses. Participants sampled within 14 days of booster or later than 50 days after booster were excluded from the boosting phase analysis. We excluded those who reported, or had immunological evidence of, SARS-CoV-2 infection, between the timepoints that make up one of the phases above. In the waning phase, we defined evidence of SARS-CoV-2 infection as anti-nucleocapsid seroconversion between the post-booster and follow-up timepoints. For those who were already seropositive for anti-nucleocapsid antibodies, we considered a higher anti-nucleocapsid antibody titre at follow-up compared to post-booster, or a higher ELISpot count in response to nucleocapsid or membrane peptides at 6-months compared to 1-month post-booster, with a concurrent rise in anti-spike antibody titre as evidence of infection.

We defined evidence of SARS-CoV-2 infection in the pre-vaccination to 1-month post-vaccination period as anti-nucleocapsid seroconversion between the two timepoints or, for those who were seropositive at the pre-booster timepoint, a fourfold increase in anti-nucleocapsid titre between timepoints. Additionally, having a twofold increase in anti-nucleocapsid titre with evidence of a higher ELISpot count in response to SARS-CoV-2 nucleocapsid or membrane peptides at the 1-month post-vaccine visit compared to the pre-vaccine visit was considered as evidence of infection. The higher thresholds for anti-nucleocapsid titre rise in the boosting phase were selected on the basis of previous work indicating that anti-nucleocapsid titres may be boosted by vaccination against SARS-CoV-2 spike protein^31^. These definitions have also been used in previous work^32^.

We also excluded those who were defined as ‘clinically extremely vulnerable’ and had received ≥ 3 vaccinations at the start of the study period.

We derived a three-level ethnicity variable (White vs. South Asian vs. Black/Mixed/Other). We acknowledge that the latter group is highly heterogeneous, however, we were keen not to exclude participants on the basis of their ethnicity and numbers of participants within each of the subgroups (Black, Mixed or Other) were too small to make meaningful comparisons with the reference group.

We summarised demographic and vaccine related parameters for those included in each analysis as frequency and percentage for categorical and median and interquartile range (IQR) for continuous variables. We compared these parameters across ethnic groups, using Kruskal-Wallis test for continuous variables and chi-squared for categorical variables to determine differences in confounding variables by ethnicity. We also examined relationships between all covariates and total SARS-CoV-2 anti-spike antibody titre at the post-booster and follow-up timepoints using univariable linear regression.

For the pseudotype neutralisation assays, an initial screen of samples was performed at a dilution of 1:50. Any sample with neutralising activity < 90% at this dilution was coded as a titre of 50 for the purposes of this analysis. Samples with a neutralising activity of 90% or above at 1:50 dilution underwent the serial dilutions described in laboratory methods above, and the mean titre of the triplicate assay was taken. Antibody titres were log (base 10) transformed prior to multivariable analysis. We subtracted the unstimulated ELISpot count from the count after stimulation with peptides and then multiplied by four to give a count per million PBMCs. We summarised unadjusted titres/ELISpot counts at each timepoint reporting geometric mean titre (GMT) and 95% confidence interval (95%CI) for antibody titres and median (IQR) for ELISpot count.

We examined the association between ethnicity and changes in antibody titre after vaccination using multivariable linear regression. Examination of the phases of the immune response described above was achieved by using either post-booster titre or follow-up titre (depending on the phase) as the dependent variable and adjusting for antibody titre at the previous timepoint and time in days between the two timepoints. Adjustment of the model for antibody response at the previous timepoint allows us to comment on change in titre between the two timepoints. We then additionally adjusted this analysis for hypothesised confounders of the association between ethnicity and immune response (age, sex, type of SARS-CoV-2 vaccines received and previous SARS-CoV-2 infection status [as defined by anti-nucleocapsid serostatus]). We also used multivariable linear regression to model the absolute and fold change in total antibody titre between the timepoints after adjustment for the same confounders excluding those with missing data. We exponentiated (base 10) the linear regression coefficients and present associations as adjusted Geometric Mean Ratios (aGMRs) and 95%CIs. To ensure that any differences in humoral response to vaccination were not driven by differing occupational exposure to SARS-CoV- 2 by ethnicity we conducted a sensitivity analysis additionally adjusting our multivariable analyses of the humoral response to vaccination for a binary measure indicating whether a HCW reported having had physical contact with COVD-19 patients in the week prior to the baseline survey. To investigate the effects of adjusting for previous infection status, as a sensitivity analysis, we also examined the relationship between ethnicity and humoral responses to vaccination in an infection naïve cohort (defined by seronegativity for anti-nucleocapsid antibody). We did not conduct this analysis in he waning cohort as the sample size after restrictions was felt to be too small.

The association between ethnicity and changes in T cell responses using ELISpot count after vaccination were modelled using multivariable negative binomial regression adjusted for the same pre-specified confounders as in the serology analyses.

All analyses were conducted using Stata 17 (StataCorp. 2021. Stata Statistical Software: Release 17. College Station, TX: StataCorp LLC.). Figures were created in GraphPad Prism version 9.4.1 (GraphPad Software, San Diego, California USA, www.graphpad.com). We considered P values < 0.05 to be statistically significant.

## Supplementary Information

Below is the link to the electronic supplementary material.

Supplementary Material 1

## Data Availability

The datasets generated and/or analysed during the current study are not publicly available due to ongoing primary analyses by the study team but are available from the corresponding author on reasonable request, subject to approval by the UK-REACH Core Management Group and Steering Committee. To access data or samples produced by the BE-DIRECT study, the working group representative must first submit a request to the Core Management Group by contacting the UK-REACH Project Manager (uk-reach@leicester.ac.uk) in the first instance. For ancillary studies outside of the core deliverables, the Steering Committee will make final decisions once they have been approved by the Core Management Group. Decisions on granting the access to data/materials will be made within eight weeks. Third party requests from outside the Project will require explicit approval of the Steering Committee once approved by the Core Management Group.
