# Supplementary material for: The impact of ethnicity on longitudinal humoral and cellular immune responses to SARS-CoV-2 booster vaccination
Source: Sci Rep. 2026 Jun 27;16:29559. doi: 10.1038/s41598-026-54217-5 (PMC13597308; doi:10.1038/s41598-026-54217-5)
Supplement: Supplementary file 1 [file 41598_2026_54217_MOESM1_ESM.docx]

**The impact of ethnicity on longitudinal humoral and cellular immune responses to SARS-CoV-2 booster vaccination**

Christopher A. Martin (MD)^1,2,3,4^, Joshu­­­­­­a Nazareth (MD)^1,2,3,4^, Lucy Teece (PhD)^5^, Daniel Pan (MRCP)^1,2,3,4,6^, Sam Scott^7^, Amar Jarkhi (PhD)^8^, Mrinal Das (PhD)^8^, Luke Bryant (PhD)^1,4^, Nisha George (MRCP)^1,2,3^, Marjan Gohar (MRCP)^1,2,3^, Valerie Renals^9^, Denny Vail^9^, Aleesha Karia^9^, Paul Moss (PhD)^10^, Brian J. Willett (PhD)^7^, Andrea Tattersall (PhD)^11^, Bassam Hallis (PhD)^12^, Ashley D. Otter^12^(PhD), Cathy Rowe^12^ (MSc), Pranab Haldar (MD)^13,14^, Andrea Cooper (PhD)^8^, Manish Pareek (PhD)^1,2,3,4,14^*

*Corresponding author [manish.pareek@leicester.ac.uk](mailto:manish.pareek@leicester.ac.uk); University of Leicester, University Road, Leicester, LE1 7RH, UK; tel: [+44 (0)116 2585506](tel:+441162585506)

1. Division of Public Health and Epidemiology, School of Medical Sciences, University of Leicester, Leicester, UK

2. Department of Infection and HIV Medicine, University Hospitals of Leicester NHS Trust, Leicester, UK

3. Development Centre for Population Health, University of Leicester, UK

4. Leicester NIHR Biomedical Research Centre, Leicester, UK

5. Department of Population Health Sciences, College of Life Sciences, University of Leicester, Leicester, UK

6. Li Ka Shing Centre for Health Information and Discovery, Oxford Big Data Institute, University of Oxford

7. University of Glasgow Centre for Virus Research, University of Glasgow, Bearsden Road, Glasgow, UK

8. Division of Microbiology and Infection, School of Biological and Biomedical Sciences, University of Leicester, Leicester, UK

9. Research Space, University Hospitals of Leicester NHS Trust

10. Institute of Immunology and Immunotherapy, University of Birmingham, Birmingham, UK

11. Revvity (Certimmune), Abingdon, Oxfordshire, UK

12. UK Health Security Agency, Porton Down, Salisbury, UK

13. Division of Respiratory Sciences, School of Medical Sciences, University of Leicester, Leicester, UK

14. Department of Respiratory Medicine, University Hospitals of Leicester NHS Trust, Glenfield Hospital, Leicester, UK

15**.** NIHR Applied Research Collaboration East Midlands, Leicester, UK

**Supplementary Table 1. Details of peptides used in ELISpot assays**

| **Panel ID** | **Description** | **Origin** |
| --- | --- | --- |
| 1 | Mix of 156 peptides  (15mers, overlap 11) | Spike Protein (S1) - N Terminus (with exclusions of areas with high homology to other organisms) of Wuhan wild type |
| 2 | Mix of 139 peptides | Spike Protein (S2) - C Terminus (with exclusions of areas with high homology to other organisms) of Wuhan wild type |
| 3 | Mix of 97 peptides  (15mers, overlap 11) | Nucleocapsid Protein of Wuhan wild type |
| 4 | Mix of 50 peptides | Membrane Protein of Wuhan wild type |

As our analysis focusses on vaccine induced immune responses (i.e. immune responses to spike proteins) we used data from the Panel 1 (S1) and Panel 2 (S2) in this analysis. Raw ELISpot counts were transformed first by subtracting the count from an unstimulated control sample and then multiplying the result by four to give a count / million PMBCs. Total spike responses were calculated by adding the S1 and S2 responses.

**Supplementary Table 2. Unadjusted linear regression models showing the univariable associations between covariates and total anti-spike antibody titres at post-booster and follow-up timepoints**

| **Variable** | **Outcome – post-booster anti-spike antibody titre** | | **Outcome – follow-up anti-spike antibody titre** | |
| --- | --- | --- | --- | --- |
|  | **GMR (95% CI)** | **P value** | **GMR (95%CI)** | **P value** |
| **Ethnicity**  White  South Asian  Black/Mixed/Other | Ref  1.45 (1.17 – 1.79)  1.08 (0.78 – 1.49) | -  0.001  0.64 | Ref  1.22 (0.84 – 1.77)  1.17 (0.61 – 2.25) | -  0.29  0.64 |
| **Time between booster and sample collection (for post-booster analysis) or time between post-booster and follow-up sample collection (for follow-up analysis)**, per week increase | 0.93 (0.86 – 1.01) | 0.09 | 0.97 (0.94 – 1.00) | 0.05 |
| **SARS-CoV-2 total anti-spike antibody titre at previous timepoint,** per 10-fold increase | 1.69 (1.48 – 1.93) | <0.001 | 9.07 (6.54 – 12.57) | <0.001 |
| **Age**, per decade increase | 0.98 (0.92 – 1.04) | 0.53 | 0.99 (0.88 – 1.12) | 0.88 |
| **Sex**  Male  Female | Ref  1.05 (0.87 – 1.27) | -  0.62 | Ref  1.05 (0.73 – 1.51) | -  0.79 |
| **Vaccine schedule**  BNT/BNT/BNT  ChAd/ChAd/BNT | -  0.85 (0.72 – 1.02) | -  0.08 | Ref  1.24 (0.73 – 1.41) | -  0.94 |
| **Anti-nucleocapsid serostatus at previous appointment**  Negative  Positive | -  1.28 (1.09 – 1.52) | -  0.004 | Ref  2.33 (1.82 – 2.98) | -  P<0.001 |

Supplementary Table 2 shows the results from univariable linear regression models to show the unadjusted associations between all covariates and total SARS-CoV-2 anti-spike antibody titres at the post-booster and follow-up timepoints

| **Variable** | **Model 1 (‘minimally adjusted’)**  **(n=291)** | | **Model 2 (‘fully adjusted’)**  **(n=290)** | |
| --- | --- | --- | --- | --- |
|  | **aGMR (95% CI)** | **P value** | **aGMR (95%CI)** | **P value** |
| **Ethnicity**  White  South Asian  Black/Mixed/Other | Ref  1.50 (1.24 – 1.82)  1.05 (0.78 – 1.40) | -  <0.001  0.76 | Ref  1.56 (1.27 – 1.90)  1.04 (0.77 – 1.40) | -  <0.001  0.80 |
| **Time between booster and sample collection**, per week increase | 0.90 (0.83 – 0.96) | 0.003 | 0.89 (0.83 – 0.96) | 0.002 |
| **Pre-booster SARS-CoV-2 total anti-spike antibody titre,** per 10-fold increase | 1.69 (1.49 – 1.92) | <0.001 | 2.01 (1.69 – 2.38) | <0.001 |
| **Age**, per decade increase | - | - | 1.01 (0.96 – 1.07) | 0.62 |
| **Sex**  Male  Female | - | - | Ref  1.05 (0.88 – 1.24) | -  0.61 |
| **Vaccine schedule**  BNT/BNT/BNT  ChAd/ChAd/BNT | - | - | Ref  1.24 (1.03 – 1.48) | -  0.02 |
| **Anti-nucleocapsid serostatus at pre-booster appointment**  Negative  Positive | - | - | Ref  0.81 (0.67 – 0.97) | -  0.03 |

**Supplementary Table 3. Linear regression models to show the relationship between ethnicity and other sociodemographic and vaccine related parameters with post-booster SARS-CoV-2 total anti-spike antibody titre**

Supplementary Table 3 shows the results of two linear regression models. The outcome is log_10_ transformed post-booster antibody titres. Coefficients were exponentiated and presented as adjusted geometric mean ratio (aGMR) and 95% confidence interval (95%CI). One participant had missing data for vaccine schedule and is therefore missing from the fully adjusted model.

**Supplementary Table 4. Linear regression models to show the relationship between ethnicity with post-booster SARS-CoV-2 total anti-spike antibody titre and SARS-CoV-2 B.1 antibody titre for 50% neutralisation in an infection naïve cohort**

| **Post-booster SARS-CoV-2 total anti-spike antibody titre** | | | | |
| --- | --- | --- | --- | --- |
| **Variable** | **Model 1 (‘minimally adjusted’)**  **(n=217)** | | **Model 2 (‘fully adjusted’)**  **(n=216)** | |
|  | **aGMR (95% CI)** | **P value** | **aGMR (95%CI)** | **P value** |
| **Ethnicity**  White  South Asian  Black/Mixed/Other | Ref  1.56 (1.23 – 1.97)  1.08 (0.74 – 1.57) | -  <0.001  0.51 | Ref  1.61 (1.27 - 2.03)  1.00 (0.68 – 1.49) | -  <0.001  0.99 |
| **SARS-CoV-2 B.1 antibody titre for 50% neutralisation** | | | | |
| **Variable** | **Model 1 (‘minimally adjusted’)**  **(n=215)** | | **Model 2 (‘fully adjusted’)**  **(n=214)** | |
|  | **aGMR (95% CI)** | **P value** | **aGMR (95%CI)** | **P value** |
| **Ethnicity**  White  South Asian  Black/Mixed/Other | Ref  1.90 (1.15 – 3.14)  1.31 (0.57 – 3.01) | -  0.012  0.52 | Ref  1.89 (1.13 – 3.14)  1.11 (0.46 – 2.71) | -  0.015  0.81 |

Supplementary Table 4 shows the results from four linear regression models. The outcomes are log_10_ transformed post-booster antibody titre in the top panel and log_10_ transformed post-booster antibody titre for 50% neutralisation of SARS-CoV-2 B.1 in the pseudotype neutralisation assay in the bottom panel. Hese analyses are restricted to those without serological evidence of infection (i.e. anti-nucleocapsid seronegative) at the post-booster timepoint. Coefficients were exponentiated and presented as adjusted geometric mean ratio (aGMR) and 95% confidence interval (95%CI). Minimally and Fully adjusted models are adjusted for the same factors as in the main analysis with the exclusion of infection status (which is omitted due to this factor defining inclusion for this subanalysis) One participant had missing data in one of the additional variables included in the fully adjusted model and is therefore missing from this analysis. Two participants were missing neutralising activity data.

**Supplementary Table 5. Linear regression models to show the relationship between ethnicity and other sociodemographic and vaccine related parameters with SARS-CoV-2 B.1 antibody titre for 50% neutralisation at the post-booster timepoint**

| **Variable** | **Model 1 (‘minimally adjusted’)**  **(n=288)** | | **Model 2 (‘fully adjusted’)**  **(n=287)** | |
| --- | --- | --- | --- | --- |
|  | **aGMR (95% CI)** | **P value** | **aGMR (95%CI)** | **P value** |
| **Ethnicity**  White  South Asian  Black/Mixed/Other | Ref  1.89 (1.27 - 2.81)  1.27 (0.69 – 2.31) | -  0.002  0.44 | Ref  1.82 (1.22 - 2.72)  1.10 (0.59 - 2.05) | -  0.004  0.77 |
| **Time between booster and sample collection**, per week increase | 0.85 (0.74 – 0.99) | 0.03 | 0.87 (0.75 – 1.00) | 0.05 |
| **Pre-booster SARS-CoV-2 B.1 antibody titre for 50% neutralisation,** per 10-fold increase | 2.05 (1.71 – 2.47) | <0.001 | 1.74 (1.36 – 2.22) | <0.001 |
| **Age**, per decade increase | - | - | 1.01 (0.90 – 1.13) | 0.86 |
| **Sex**  Male  Female | - | - | Ref  1.08 (0.76 – 1.53) | -  0.67 |
| **Vaccine schedule**  BNT/BNT/BNT  ChAd/ChAd/BNT | - | - | Ref  0.96 (0.69 – 1.33) | -  0.80 |
| **Anti-nucleocapsid serostatus at pre-booster appointment**  Negative  Positive | - | - | Ref  1.57 (1.04 – 2.35) | -  0.03 |

Supplementary Table 5 shows the results of two linear regression models. The outcome is log_10_ transformed post-booster antibody titre for 50% neutralisation of SARS-CoV-2 B.1 in the pseudotype neutralisation assay. Coefficients were exponentiated and presented as adjusted geometric mean ratio (aGMR) and 95% confidence interval (95%CI). Three patients included in the total antibody analyses had missing data for neutralisation titres and are missing from this analysis. One participant had missing data in one of the additional variables included in the fully adjusted model and is therefore missing from this analysis.

**Supplementary Table 6. Linear regression models to show the relationship between ethnicity and other sociodemographic and vaccine related parameters with absolute and fold change in antibody titres between the pre-boost and post-boost timepoints**

| **Variable** | **Absolute change (minimally adjusted)**  **(n=291)** | | **Absolute change (fully adjusted)**  **(n=290)** | | **Fold change (minimally adjusted)**  **(n=291)** | | **Fold change (fully**  **adjusted)**  **(n=290)** | |
| --- | --- | --- | --- | --- | --- | --- | --- | --- |
|  | **Adjusted ratio** | **P value** | **Adjusted ratio** | **P value** | **Adjusted ratio** | **P value** | **Adjusted ratio** | **P value** |
| **Ethnicity**  White  South Asian  Black/Mixed/Other | Ref  1.64 (1.30 – 2.07)  1.07 (0.75 – 1.51) | -  <0.001  0.72 | Ref  1.62 (1.28 – 2.05)  1.01 (0.71 – 1.46) | -  <0.001  0.94 | Ref  1.42 (0.99 – 2.06)  0.85 (0.49 – 1.46) | -  0.06  0.55 | Ref  1.80 (1.35 – 2.40)  1.09 (0.70 – 1.69) | -  <0.001  0.72 |
| **Time between post-booster and follow-up sample collection**, per week increase | 0.86 (0.77 – 0.93) | <0.001 | 0.86 (0.79 – 0.94) | 0.001 | 0.86 (0.75 – 0.99) | 0.03 | 0.86 (0.78 – 0.96) | 0.006 |
| **Age**, per decade increase | - | - | 1.00 (0.94 – 1.07) | 0.91 | - | - | 1.06 (0.98 – 1.15) | 0.15 |
| **Sex**  Male  Female | - | - | Ref  1.04 (0.84 – 1.28) | -  0.72 | - | - | Ref  1.09 (0.85 – 1.41) | -  0.49 |
| **Vaccine schedule**  BNT/BNT/BNT  ChAd/ChAd/BNT | - | - | Ref  0.88 (0.73 – 1.07) | -  0.20 | - | - | Ref  2.84 (2.25 – 3.59) | -  <0.001 |
| **Anti-nucleocapsid serostatus at pre-booster appointment**  Negative  Positive | - | - | Ref  0.98 (0.82 – 1.18) | -  0.85 | - | - | Ref  0.28 (0.22 – 0.35) | -  <0.001 |

Supplementary Table 6 shows the results of two linear regression models. The outcomes are log_10_ absolute change in antibody titre (i.e. log_10_ (post-booster titre – pre-booster titre)) and log_10_ fold change in antibody titre (i.e. log_10_ post-booster titre - log_10_ pre-booster titre). Coefficients were exponentiated and presented as an adjusted ratio representing the degree of change in antibody titres between the timepoints.

**Supplementary Table 7. Linear regression models to show the relationship between ethnicity and other sociodemographic and vaccine related parameters with follow-up (approx. 6 month post booster) SARS-CoV-2 total anti-spike antibody titre**

Supplementary Table 7 shows the results of two linear regression models. The outcome is log_10_ transformed follow-up antibody titres. Coefficients were exponentiated and presented as adjusted geometric mean ratio (aGMR) and 95% confidence interval (95%CI). Two

| **Variable** | **Model 1 (‘minimally adjusted’)**  **(n=174)** | | **Model 2 (‘fully adjusted’)**  **(n=172)** | |
| --- | --- | --- | --- | --- |
|  | **aGMR (95% CI)** | **P value** | **aGMR (95%CI)** | **P value** |
| **Ethnicity**  White  South Asian  Black/Mixed/Other | Ref  0.90 (0.68 – 1.18)  1.19 (0.77 – 1.83) | -  0.43  0.43 | Ref  0.85 (0.66 – 1.09)  1.35 (0.88 – 2.08) | -  0.20  0.17 |
| **Time between post-booster and follow-up sample collection**, per day increase | 1.00 (0.99 – 1.00) | 0.03 | 1.00 (0.99 – 1.00) | 0.02 |
| **Post-booster SARS-CoV-2 total anti-spike antibody titre,** per 10-fold increase | 9.30 (6.70 – 12.93) | <0.001 | 7.48 (5.41 – 10.34) | <0.001 |
| **Age**, per decade increase | - | - | 1.01 (0.93 – 1.09) | 0.84 |
| **Sex**  Male  Female | - | - | Ref  0.91 (0.72 – 1.15) | -  0.42 |
| **Vaccine schedule**  BNT/BNT/BNT  ChAd/ChAd/BNT | - | - | Ref  1.06 (0.85 – 1.32) | -  0.62 |
| **Anti-nucleocapsid serostatus at post-booster appointment**  Negative  Positive | - | - | Ref  1.67 (1.38 – 2.01) | -  <0.001 |

participants were missing data in the additional variables included in the ‘fully adjusted’ model and are therefore missing from this analysis.

**Supplementary Table 8. Linear regression models to show the relationship between ethnicity and other sociodemographic and vaccine related parameters with SARS-CoV-2 B.1 antibody titre for 50% neutralisation at the follow-up timepoint**

| **Variable** | **Model 1 (‘minimally adjusted’)**  **(n=172)** | | **Model 2 (‘fully adjusted’)**  **(n=170)** | |
| --- | --- | --- | --- | --- |
|  | **aGMR (95% CI)** | **P value** | **aGMR (95%CI)** | **P value** |
| **Ethnicity**  White  South Asian  Black/Mixed/Other | Ref  1.45 (0.74 – 2.84)  1.48 (0.92 – 2.36) | -  0.27  0.10 | Ref  1.17 (0.64 – 2.15)  2.64 (0.93 – 7.53) | -  0.61  0.07 |
| **Time between post-booster and follow-up sample collection**, per day increase | 0.99 (0.94 – 1.05) | 0.80 | 1.00 (0.95 – 1.05) | 0.91 |
| **Post-booster SARS-CoV-2 total anti-spike antibody titre,** per 10-fold increase | 4.56 (3.05 – 6.82) | <0.001 | 2.69 (1.81 – 4.00) | <0.001 |
| **Age**, per decade increase | - | - | 1.00 (0.82 – 1.21) | 0.99 |
| **Sex**  Male  Female | - | - | Ref  0.92 (0.52 – 1.65) | -  0.79 |
| **Vaccine schedule**  BNT/BNT/BNT  ChAd/ChAd/BNT | - | - | Ref  1.31 (0.76 – 2.23) | -  0.33 |
| **Anti-nucleocapsid serostatus at post-booster appointment**  Negative  Positive | - | - | Ref  4.96 (3.06 – 8.05) | -  <0.001 |

Supplementary Table 8 shows the results of two linear regression models. The outcome is log_10_ transformed antibody titre for 50% neutralisation of SARS-CoV-2 B.1 in the pseudotype neutralisation assay at the follow-up timepoint (approx.. 6 months after booster vaccine).Coefficients were exponentiated and presented as adjusted geometric mean ratio (aGMR) and 95% confidence interval (95%CI). Two participants including in the total antibody analysis were missing data for neutralising titres and two participants were missing data in the additional variables included in the ‘fully adjusted’ model and are therefore missing from this analysis.

**Supplementary Table 9. Linear regression models to show the relationship between ethnicity and other sociodemographic and vaccine related parameters with absolute and fold change in SARS-CoV-2 total antibody titres between the post-boost and follow-up timepoints**

| **Variable** | **Absolute change (minimally adjusted)**  **(n=172)** | | **Absolute change (fully adjusted)**  **(n=170)** | | **Fold change (minimally adjusted)**  **(n=174)** | | **Fold change (fully**  **adjusted)**  **(n=172)** | |
| --- | --- | --- | --- | --- | --- | --- | --- | --- |
|  | **Adjusted ratio** | **P value** | **Adjusted ratio** | **P value** | **Adjusted ratio** | **P value** | **Adjusted ratio** | **P value** |
| **Ethnicity**  White  South Asian  Black/Mixed/Other | Ref  1.57 (1.18 – 2.09)  1.30 (0.80 – 2.12) | -  0.002  0.28 | Ref  1.60 (1.20 – 2.13)  1.15 (0.69 – 1.94) | -  0.001  0.59 | Ref  1.13 (0.87 – 1.47)  0.84 (0.55 – 1.29) | -  0.36  0.42 | Ref  1.24 (0.97 – 1.60)  0.73 (0.47 – 1.12) | -  0.09  0.14 |
| **Time between post-booster and follow-up sample collection**, per day increase | 1.00 (1.00 – 1.00) | 0.55 | 1.00 (1.00 – 1.00) | 0.35 | 1.00 (1.00 – 1.01) | 0.03 | 1.00 (1.00 – 1.01) | 0.03 |
| **Age**, per decade increase | - | - | 1.03 (0.94 – 1.13) | 0.52 | - | - | 1.00 (0.92 – 1.08) | 0.97 |
| **Sex**  Male  Female | - | - | Ref  1.07 (0.82 – 1.40) | -  0.61 | - | - | Ref  1.11 (0.87 – 1.40) | -  0.40 |
| **Vaccine schedule**  BNT/BNT/BNT  Chad/Chad/BNT | - | - | Ref  0.73 (0.57 – 0.94) | -  0.01 | - | - | Ref  0.92 (0.74 – 1.14) | -  0.45 |
| **Anti-nucleocapsid serostatus at post-booster appointment**  Negative  Positive | - | - | Ref  1.20 (0.98 – 1.48) | -  0.08 | - | - | Ref  0.63 (0.53 – 0.76) | -  <0.001 |

Supplementary Table 9 shows the results of four linear regression models. The outcomes are log_10_ absolute change in antibody titre (i.e. log_10_ (post-booster titre – pre-booster titre)) and log_10_ fold change in antibody titre (i.e. log_10_ post-booster titre - log_10_ pre-booster titre). Coefficients were exponentiated and presented as an adjusted ratio representing the degree of change in antibody titres between the timepoints.

**Supplementary Table 10. Additional adjustment for reported degree of exposure to patients with confirmed COVID-19 in each phase of the humoral response to vaccination**

| **Boosting phase** | | | | | | | | |
| --- | --- | --- | --- | --- | --- | --- | --- | --- |
| **Variable** | **Total SARS-CoV-2 anti-spike antibody titre** | | | | **SARS-CoV-2 B.1 antibody titre for 50% neutralisation** | | | |
|  | **aGMR (95% CI)** | | **P value** | | **aGMR (95%CI)** | | | **P value** |
| **Ethnicity**  White  South Asian  Black/Mixed/Other | Ref  1.51 (1.24 – 1.84)  1.04 (0.78 – 1.40) | | -  <0.001  0.10 | | Ref  1.74 (1.16 – 2.62)  1.10 (0.59 – 2.06) | | | -  0.008  0.77 |
| **Waning phase** | | | | | | | | |
| **Ethnicity**  White  South Asian  Black/Mixed/Other | | Ref  0.86 (0.66 – 1.11)  1.34 (0.87 – 2.06) | | -  0.25  0.19 | | Ref  1.25 (0.67 – 2.34)  2.54 (0.89 – 7.23) | -  0.48  0.08 | |

Supplementary Table 10 shows the relationship between ethnicity and change in antibody titres after booster vaccination using linear regression models similar to those shown above in the fully adjusted model. These models have been additionally adjusted for a binary variable indicating whether a participant indicated they had physical contact with confirmed COVID-19 patients or not in the baseline questionnaire. Coefficients were exponentiated and presented as adjusted geometric mean ratio (aGMR) and 95% confidence interval (95%CI).

**Supplementary Table 10. Description of ELISpot counts by ethnicity**

| **Phase** | **Timepoint** | **White**  **Boosting phase n=229**  **Waning phase n=136** | | | **South Asian**  **Boosting phase n=38**  **Waning phase n=28** | | | **Black/Mixed/Other**  **Boosting phase n=14**  **Waning phase n=8** | | |
| --- | --- | --- | --- | --- | --- | --- | --- | --- | --- | --- |
|  |  | **S1**  **med(IQR)** | **S2**  **med(IQR)** | **Spike**  **med(IQR)** | **S1**  **med(IQR)** | **S2**  **med(IQR)** | **Spike**  **med(IQR)** | **S1**  **med(IQR)** | **S2**  **med(IQR)** | **Spike**  **med(IQR)** |
| **Boosting phase** | **Pre-booster** | 3 (1- 8) | 3 (1-6) | 6 (3-14) | 4 ( 1 – 10) | 4 (1 – 9) | 9 (3 – 26) | 4 (1 – 9) | 3 (0 – 10) | 6 (2 – 20) |
|  | **Post-booster** | 12 (5 – 25) | 8 (3 – 17) | 22 (10 – 45) | 11 (4 – 31) | 8 (5 – 15) | 20 (11 – 48) | 13 ( 2 – 18) | 10 (3 – 26) | 25 (5 – 36) |
| **Waning**  **phase** | **Post-booster** | 14 (6 – 30) | 9 (4 – 18) | 24 (12 – 56) | 10 (5.5 – 21) | 7.5 (6 – 14) | 22.5 (11 – 31) | 18 (10 – 29) | 13 (12 – 23) | 36 (25 – 43) |
|  | **Follow-up** | 4 (1 – 12) | 3 (1 – 7) | 7 (3 – 20) | 4 (2 – 10.5) | 5 (2.5 – 9)* | 10 (5.5 – 16.5) | 6 (4 – 8) | 4 (2 – 5) | 11 (9 – 15) |

*p = 0.04 vs White by Wilcoxon rank-sum

Supplementary Table 10 shows a description of the ELISpot counts (in SFU/250,000 PBMCs) at each timepoint and by each phase of the immune response stratified by ethnic group. Wilcoxon rank-sum tests were used to compare ELISpot counts between ethnic groups at each time point. The one significant difference by ethnicity is indicated by the asterisk

**Supplementary Figure 1. Comparison of unadjusted ELISpot count by ethnicity during each phase of the immune response**

Supplementary Figure 1 shows unadjusted ELISpot counts in response to stimulation with SARS-CoV-2 spike peptides (S1, S2 and spike [S1+S2]). Black bars indicate the median. Responses are shown numerically and compared in Supplementary Table 7.
